# Does prior dengue virus exposure worsen clinical outcomes of Zika virus infection? A systematic review, pooled analysis and lessons learned

**DOI:** 10.1371/journal.pntd.0007060

**Published:** 2019-01-25

**Authors:** Jennifer Masel, Michael K. McCracken, Todd Gleeson, Brian Morrison, George Rutherford, Allison Imrie, Richard G. Jarman, Michael Koren, Simon Pollett

**Affiliations:** 1 Department of Medicine, USUHS, Bethesda, MD, United States of America; 2 Viral Diseases Branch, Walter Reed Army Institute of Research, Silver Spring, MD, United States of America; 3 Institute for Global Health Sciences, University of California, San Francisco, CA, United States of America; 4 University of Western Australia, Perth, WA, Australia; 5 Department of Preventive Medicine & Biostatistics, USUHS, Bethesda, MD, United States of America; 6 Marie Bashir Institute for Infectious Diseases, University of Sydney, Camperdown, NSW, Australia; George Washington University School of Medicine and Health Sciences, UNITED STATES

## Abstract

Zika virus (ZIKV) recently caused a pandemic complicated by Guillain-Barre syndrome (GBS) and birth defects. ZIKV is structurally similar to the dengue viruses (DENV) and *in vitro* studies suggest antibody dependent enhancement occurs in ZIKV infections preceded by DENV; however, the clinical significance of this remains unclear. We undertook a PRISMA-adherent systematic review of all current human and non-human primate (NHP) data to determine if prior infection with DENV, compared to DENV-naïve hosts, is associated with a greater risk of ZIKV clinical complications or greater ZIKV peak viremia *in vivo*. We identified 1146 studies in MEDLINE, EMBASE and the grey literature, of which five studies were eligible. One human study indicated no increase in the risk of GBS in ZIKV infections with prior DENV exposure. Two additional human studies showed a small increase in ZIKV viremia in those with prior DENV exposure; however, this was not statistically significant nor was it associated with an increase in clinical severity or adverse pregnancy outcomes. While no meta-analysis was possible using human data, a pooled analysis of the two NHP studies leveraging extended data provided only weak evidence of a 0.39 log10 GE/mL rise in ZIKV viremia in DENV experienced rhesus macaques compared to those with no DENV exposure (p = 0.22). Using a customized quality grading criteria, we further show that no existing published human studies have offered high quality measurement of both acute ZIKV and antecedent DENV infections. In conclusion, limited human and NHP studies indicate a small and non-statistically significant increase in ZIKV viremia in DENV-experienced versus DENV-naïve hosts; however, there is no evidence that even a possible small increase in ZIKV viremia would correlate with a change in ZIKV clinical phenotype. More data derived from larger sample sizes and improved sero-assays are needed to resolve this question, which has major relevance for clinical prognosis and vaccine design.

## Author summary

Zika virus (ZIKV) is a mosquito borne virus that recently caused a large epidemic with some cases complicated by ascending paralysis (Guillain-Barre syndrome) and birth defects. One major concern is that such complications may be more common in those who have had previous infection with the closely related mosquito-borne dengue virus (DENV) which also circulates in the tropics. Here, we undertook a thorough, structured review of all human and high-order animal literature to synthesize the current evidence about whether ZIKV outcomes are worse in those with previous DENV exposure. We further undertook a pooled analysis across the two major non-human primate studies to improve statistical power. We summarize that, in humans and in non-human primates, prior DENV exposure may lead to a small increase in ZIKV viral load during infection. However, we do not show that any possible increase in ZIKV viral replication is associated with a higher rate of Zika complications or Zika clinical severity. We further graded the quality of these published literature and indicate that substantial improvements in the immunological measurement of ZIKV and DENV exposure in humans are needed to answer these and other pertinent questions about these two epidemic pathogens.

## Introduction

Zika virus is a flavivirus primarily transmitted by *Aedes aegypti* mosquitos and is the causative agent of a recent global outbreak of exanthematous febrile illness complicated by Guillain-Barre syndrome (GBS), microcephaly and other birth defects [1]. ZIKV was first discovered in Uganda in 1947 and was subsequently noted to cause sporadic human infections throughout sub-Saharan Africa and in parts of Asia [2, 3]. Sixty years after its initial discovery its epidemic potential became apparent during a major outbreak in the Yap Islands of Micronesia [4]. The 2007 and 2013–2014 outbreaks in the Yap Islands and French Polynesia, respectively, were typified by self-limiting fever, rash, arthralgia, headache, and malaise or entirely asymptomatic infection [4, 5]. In 2015 and 2016 an explosive ZIKV outbreak spread through South America, Central America, and the Caribbean in a large epidemic driven by a ZIKV-naïve population and widespread abundance of *Aedes aegypti* [6].

During the Latin America epidemic, and in retrospect during the French Polynesia outbreak, it became evident that ZIKV was associated with GBS and congenital Zika syndrome (CZS) [5, 7]. Congenital infections were associated with severe neurologic outcomes, notably fetal microcephaly, but also early pregnancy loss, stillbirth, ocular abnormalities, hearing loss, limb deformities, central nervous system lesions, and growth restriction [8]. In addition to isolated case reports as early as 2011, it was also observed that ZIKV infection could be transmitted sexually during the neotropical epidemic [9–12]. Although the ZIKV pandemic has now waned in the Americas [13], there are concerns about the risk of subsequent epidemics in Asian regions, some of which have already evidently experienced long term ZIKV circulation as well as substantial recent outbreaks, in addition to the risk to neotropical regions upon replenishment of ZIKV-susceptible birth cohorts [14–16].

An especially striking feature of the most recent ZIKV outbreak is the breadth of phenotypes, ranging from asymptomatic infection through to severe complications such as GBS and CZS [17]. Investigators studying ZIKV noted that the virus shares its vector and geographic range with the dengue viruses (DENV), a related serocomplex of flaviviruses [18]. For example, geographic regions of Northern Brazil that experienced substantial ZIKV case burdens are also known to have a DENV seroprevalence exceeding 90% in adults [19]. Sequential DENV infections with different serotypes are associated with more severe disease than infections in DENV immune patients. This is believed by many investigators to be due to an immunologic phenomenon called antibody-dependent enhancement (ADE), in which cross-reactive non-neutralizing antibodies promote viral-antibody complex binding to Fcγ receptors on monocytes, thereby facilitating viral entry and leading to increased viremia [20]. ZIKV and DENV have substantial proteomic homology, and the humoral immunological interactions between these two viruses have been studied in order to better understand the more severe manifestations of ZIKV, in particular the cross reactivity of antibodies generated by DENV exposure [21, 22].

Early in the ZIKV Americas epidemic, several *in vitro* and murine studies suggested that prior DENV infection may not only fail to cross-neutralize ZIKV but also may lead to ZIKV ADE, greater ZIKV viremia and, therefore, perhaps increased clinical morbidity upon subsequent ZIKV exposure. Priyamvada et al showed that both DENV-exposed sera and derived plasmablast monoclonal antibodies from DENV-infected individuals in Thailand enhanced *in vitro* ZIKV infection in the U937 cell lines [23]. Dejnirattisai et al noted that DENV experienced human sera collected from Thailand potently cross-reacted, poorly neutralized and induced ZIKV ADE in ZIKV PF13 or HD78788 inoculated U937 myeloid cell lines [24]. Castanha et al found that sera from DENV-3 exposed humans led to the enhanced replication of ZIKV PE/243 in FcγRII-expressing K562 cell lines [19]. Bardinha et al also showed in vitro evidence of DENV-induced ZIKV ADE using Puerto Rican DENV experienced donor sera in ZIKV infected K562 cell lines, and further showed evidence that this was IgG mediated [25]. Bardinha et al extended these *in vitro* data with an immunodeficient (Stat2−/−) mouse model and showed that plasma from DENV-experienced human hosts led to increased morbidity and paralysis in PRVABC59 ZIKV infections compared to those that received control plasma. These findings were in contrast to earlier *in vitro* data which indicated that cross-reactive DENV antibodies from human natural infections actually neutralized a range of ZIKV strains [26].

Since these early studies, which generated alarm in the public media as well as scientific debate [27–29], there has been no systematic review or meta-analysis that has synthesized all available in vivo human and higher order animal evidence and provided a current consensus on whether prior DENV infection is indeed a risk factor for worsened ZIKV clinical outcomes such as GBS, CZS or whether prior DENV exposure changes possible laboratory proxies of severity such as ZIKV viremia or inflammatory cytokine profiles. Resolving this question is critical for both clinical prognosis of those with ZIKV infection as well as DENV vaccine implementation in areas endemic to both ZIKV and DENV [28].

We therefore undertook a systematic review to determine if prior infection with DENV, as compared to those with no prior DENV infection, is associated with a greater risk of ZIKV complications (including neurological and teratogenic outcomes), greater ZIKV peak viremia, greater area-under-the-curve (AUC) of viremia or other putative laboratory proxies of ZIKV severity. While a meta-analysis of human data was not possible, we extended this systematic review with a pooled analysis leveraging previously unreported extended data from a recent non-human primate (NHP) study [30]. We also graded the quality of existing studies, in particular the validity of how acute ZIKV and prior DENV exposure were measured. Collectively, our findings offer a timely update in the current understanding of how clinical ZIKV outcomes are changed by prior DENV exposure, emphasize caution in how *in vitro* and small animal data should be interpreted during emerging viral epidemics, and underscore the urgent need for improved sero-diagnostic methods in areas experiencing syndemics of DENV, ZIKV and other medically important flaviviruses.

## Methods

We followed PRISMA best practice guidelines [31]. The following eligibility criteria were developed *a priori* by the study team of eight reviewers, although the following protocol was not registered:

### Inclusion criteria

We included all human or non-human primate (NHP) studies in which the outcomes of those with ZIKV infection preceded by DENV exposure, was compared to the outcomes of those with ZIKV infection without prior DENV infection. ZIKV infection was defined as molecular detection in blood, urine, or any other biological human specimen, and/or positive serology consistent with acute infection. DENV exposure was defined as a documented prior clinically or laboratory diagnosed DENV infection, known experimental inoculation with DENV (for example, in NHPs), or serology consistent with prior DENV exposure.

We included studies which measured any of the following outcomes: magnitude of peak viremia/RNAemia, duration of viremia/RNAemia, area-under-the-curve of viremia/RNAemia, cytokine profiles, relative increase in inflammatory biomarkers in conjunction with non-neutralizing antibodies or other laboratory evidence of Zika severity, fetal microcephaly, fetal loss or other malformation, CZS, GBS, meningitis, encephalitis, myelitis, other resultant neurologic morbidity, hospitalization, and death. We included studies from any time period, and in any language.

### Exclusion criteria

We excluded studies of animals other than NHPs, *in vitro* studies, case reports, case series, book chapters, dissertations, editorials, duplicate studies, and review articles unless they contained new data and analyses.

### Literature search methods

The PubMed/MEDLINE, EMBASE, Web of Science and Scopus databases were searched on March 25, 2018 using a pre-specified search strategy (see Appendix for an example search ontology). We also searched the bioRxiv pre-print repository [32] in addition to bibliographies of recent comprehensive narrative reviews relevant to the research question [33–36], field expert consultation and review of conference abstract handbooks from IDWeek and the American Society of Tropical Medicine & Hygiene 2016–2017 [37–40].

### Determination of study eligibility

Two reviewers collated de-duplicated literature search results to EndNote in preparation for screening before each reviewer independently screened each study title and abstract for potential eligibility. Any discrepancies between the screening determinations were resolved between the reviewers by consensus, with a third reviewer adjudicating any residual disagreements. For those studies, which were screened in as potentially eligible, a reviewer pair determined final eligibility with an independent review of the full texts. Uncertainty or discrepancy between the reviewers as to whether a study met final eligibility criteria was resolved by consensus discussion, and, if necessary, a third reviewer adjudication. The reasons for excluding studies was documented.

### Data abstraction

A standardized data abstraction tool in Excel format was developed for use by a reviewer pair to extract data from the full texts of eligible studies with double-data-abstraction. Data regarding population, study type, sample size, laboratory assays, timing of laboratory testing, clinical outcomes, and other characteristics were abstracted by each reviewer in the reviewer pair. Agreement between reviewer pairs was determined and discrepancies resolved in data entry.

### Study quality assessment

Study quality was evaluated using the same two-reviewer pair system described above, following the quality grading criteria presented in S1 Table, a customized grading system developed by the review team. We provided guided examples of evaluating the quality of how prior dengue exposure and current ZIKV infection was determined (S1 Table). No review investigator graded the quality of eligible studies on which they were authors.

### Qualitative and quantitative synthesis

Qualitative data about the individual study objectives, study design, population and outcome characteristics were presented descriptively. The approach to meta-analysis or individual study participant pooled analysis was adaptive and informed by the final number of eligible studies measuring similar outcomes in a similar way (see Results). All statistical analyses were be performed using Stata version 12.0 (StataCorp, College Station, TX).

## Results

**Fig 1:**
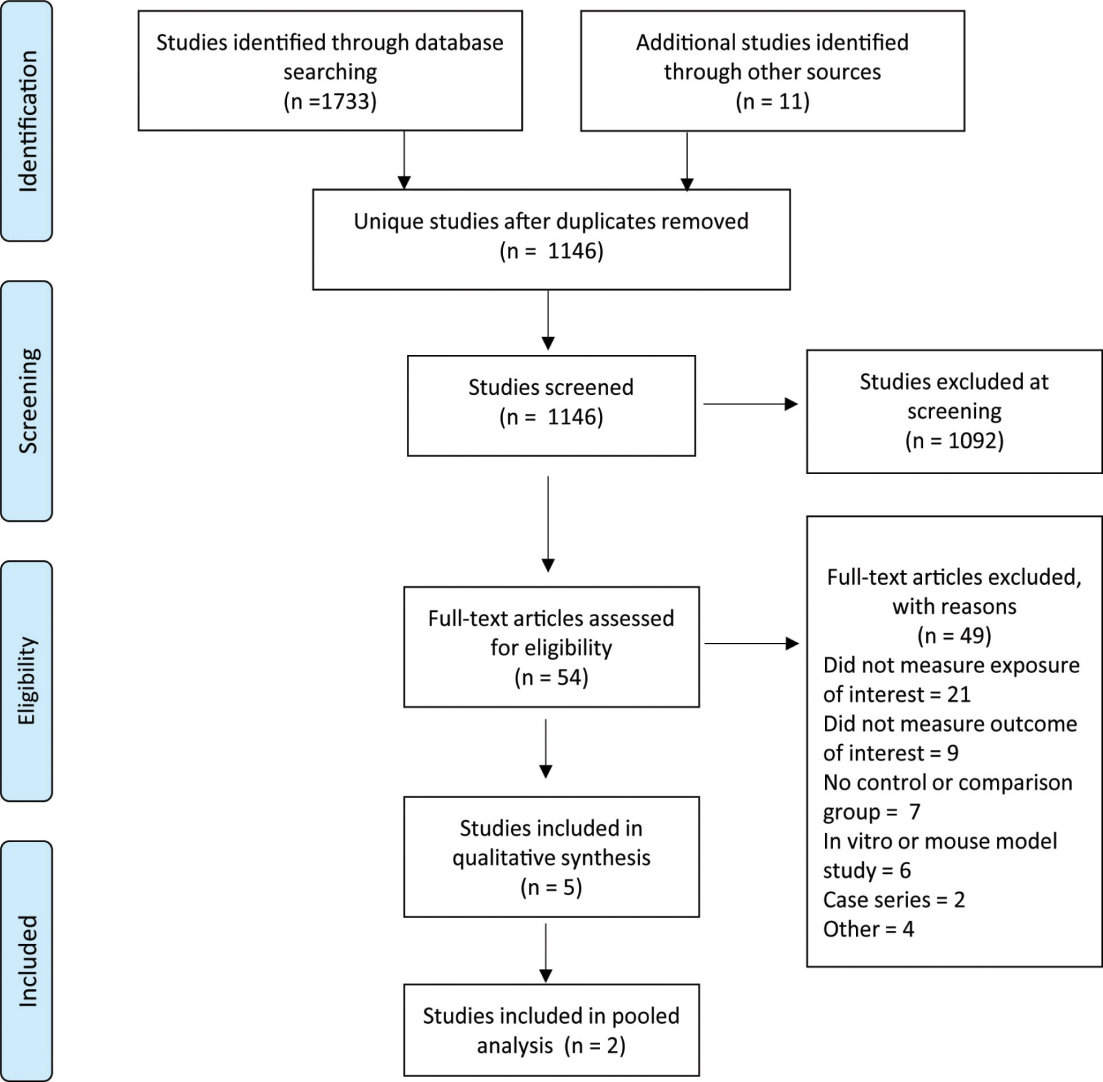
PRISMA flow-chart.

Database and grey literature searches resulted in 1146 non-duplicate citations (see Fig 1). We initially identified 54 full-text articles potentially meeting inclusion criteria, and, of these, five studies met inclusion criteria and were graded for quality according to the pre-agreed criteria (S1 Table) [5, 30, 41–43]. These studies included three human studies and two studies of NHPs. Results of the quality grading for these five studies are summarized in Table 1.

**Table 1:** Quality of eligible studies.

Study first author and citation	Was the population, intervention, comparator group and outcome described clearly?	Were the study methods presented in a reproducible way?	Was prior DENV exposure measured in a valid way?	Was acute ZIKV exposure measured in a valid way?	Were clinical outcomes and/or their laboratory proxies measured in a valid way?
Terzian	H	H	L	H	H
Cao-Lormeau	H	H	M	M	H
Halai	H	H	L	H	H
McCracken	H	H	H	H	H
Pantoja	H	H	H	H	H

L = low to moderate; M = moderate to high; H = high-very high.

### Human studies

Cao-Lormeau et al presented a case-control study of patients with ZIKV-associated GBS during the 2013–2014 French Polynesia ZIKV outbreak, which coincided with a substantial increase in the incidence of GBS and an apparent co-circulation of DENV serotypes 1 and 3 in French Polynesia [5]. The GBS case group consisted of 42 cases presenting to hospital care and diagnosed by international diagnostic criteria. A first control group (CTR1, n = 98) consisted of age, gender and residence-matched individuals presenting for clinical care with a non-febrile illness. A second control group (CTR2, n = 70) consisted of age-matched patients with RT-PCR confirmed acute ZIKV infection with no neurological complications. The investigators measured DENV and ZIKV exposure using several assays, not all of which were used in all groups. In summary, a single acute specimen from the GBS and CTR2 group underwent testing for ZIKV using a RT-PCR assay. A single acute specimen from the GBS and CTR1 group was tested for DENV and ZIKV IgM using an indirect immunofluorescence assay. Single acute specimens from the GBS, CTR1 and CTR2 groups were tested for ZIKV and DENV IgG using a recombinant-antigen based microsphere immunoassay. In addition, single acute specimens from the GBS and CTR1 groups were tested for ZIKV and DENV neutralizing antibodies using a microseroneutralization assay. While serial ZIKV and DENV serology was performed in the GBS group, logistic regression analysis to determine whether prior DENV exposure was more likely in ZIKV+/GBS+ cases compared to ZIKV+/GBS- cases apparently only used a single time-point serological measurement.

None of the GBS group were ZIKV RT-PCR positive (0 of the 41 tested from the n = 42 group). 73.8% of the GBS group had anti-ZIKV IgM and were also anti-DENV IgM negative. By combining these ZIKV IgM results with the ZIKV IgG results, the authors determined that 97.6% of the GBS group were ZIKV-exposed. When tested by the microneutralization assay, 100% of the GBS group were deemed ZIKV exposed. Of this GBS+/ZIKV+ group (n = 42), the majority (40/42 or 95.2%) were DENV IgG+. No statistically significant difference in DENV IgG+ positivity was observed in GBS cases compared with control group 1 which had 87/98 (88.8%) DENV IgG+ individuals (OR = 2.0; 95% CI = 0.4–19.9; p = 0.62). Similarly, no statistically significant difference was observed in DENV IgG+ GBS cases when compared with the CTR2 group that was comprised of 58/70 (82.9%) DENV IgG+ individuals (OR = 6.04; 95% CI = 0.81–269.5; p = 0.10).

Terzian et al described ZIKV viral load and cytokine responses in patients presenting with acute ZIKV infection in Sao Paulo, Brazil in early 2016 [41]. They sampled 45 patients with acute ZIKV infection at a single time point, with acute ZIKV infection determined by testing for ZIKV, DENV and CHIKV by RT-PCR. Prior DENV status was ascertained by a commercial DENV IgG ELISA assay. ZIKV viral load was measured by quantitative PCR. Cytokine profiles in acute ZIKV infections were estimated using a multiplex bead analysis assay which measured interleukin (IL) 1β, IL-2, IL-4, IL-6, IL-8, IL-9, IL-10, IL-13, and IL-17. Of those 45 patients with molecularly confirmed ZIKV infection, 35 were DENV IgG positive and 10 were DENV IgG negative, indicating the majority had prior DENV exposure.

Those acute ZIKV infections which were DENV IgG+ did have higher median viral load than those who were DENV IgG-, but the difference in median VL (log10 PFU/ml) between DENV IgG +/- subjects was small (“least significant difference of 0.7 log PFU/ml” according to the authors) and did not meet statistical significance (p = 0.25). The authors commented that this study was adequately powered to detect a greater than 10-fold difference in ZIKV viremia between the 2 groups. While there was evidence that IL-1β activation was correlated with ZIKV viral load, there was no statistically significant difference in median IL-1β or any other measured cytokine levels between those ZIKV-PCR+/DENVIgG+ and ZIKV-PCR+/DENVIgG- groups. The authors concluded that this study was underpowered to detect such differences. The authors demonstrated higher median IL-1β, IL-6, IL-8 and IL-9 levels in a comparative analysis of acute PCR-confirmed DENV infections who were DENV IgG- compared to those who were DENV IgG positive (with a statistical significance corresponding to p-values of 0.0039, 0.0133, 0.0477 and 0.0391, respectively) [41]. These cytokine findings are hard to interpret as they were solely measured at a single point of time and therefore may not account for within-host correlation of the data.

Halai et al studied 131 pregnant women with acute ZIKV infection, as part of a prospective cohort of 345 pregnant women presenting for medical care in Rio de Janeiro with new onset rash. ZIKV infection was confirmed by RT-PCR on serum and/or urine. A standardized ZIKV clinical severity score was developed and applied. The score was based on severity of rash, duration of illness, duration of fever, and multisystem involvement. DENV status was ascertained by IgG ELISA. ZIKV viremia was measured by PCR cycle threshold (CT) and women were followed for birth outcomes as measured by clinical neurological assessment, fetal loss and fetal imaging abnormalities. The majority of participants were enrolled in the second trimester of pregnancy, and adverse birth outcomes occurred in 46.4% of those ZIKV infected participants with available birth outcome data. Prior DENV exposure was measured by a single specimen obtained at the time of acute ZIKV infection using a commercial DENV IgG serological assay.

Median ZIKV RNA by PCR CT was 31 in DENV IgG+ women and 32 for DENV IgG- (that is, higher viremia in the DENV IgG+ group); however, this difference was not statistically significant (p = 0.153). In those positive for DENV IgG, the median clinical severity score was 7 (moderate severity) and this was not statistically significant from the median clinical severity score of 7.5 in the DENV IgG negative group (p = 0.166). Additionally, being positive for DENV IgG+ was not associated with adverse birth outcomes (p = 0.667, OR 0.78, CI 0.255–2.397) [42].

The two human studies where ZIKV viral loads were described (Terzian et al and Halai et al) could not be combined for meta-analysis because the investigators used non-comparable units to measure the outcome (PFU/ml versus PCR CT values).

### Non-human primate studies

Two NHP studies met inclusion criteria. McCracken et al described ZIKV viremia and RNAemia in six rhesus macaques previously experimentally infected with DENV in addition to 14 rhesus macaques groups who were flavivirus naïve, as determined by seronegativity to DENV, ZIKV, Japanese encephalitis virus (JEV), West Nile Virus (WNV) and yellow fever virus (YFV)[30]. In the DENV-exposed group, DENV exposure occurred >420 days prior to experimental Brazil-ZIKV2015 infection. Importantly, the investigators confirmed DENV-experienced NHP sera obtained day 0 before NHP ZIKV inoculation recapitulated ADE ZIKV infection profiles in vitro in FcγR-bearing U937 and K562 cell lines. After ZIKV inoculation, serial sera and plasma were collected for ten days and ZIKV viremia/RNAemia was measured by RT-qPCR assay. No statistically significant differences in magnitude or duration of ZIKV viremia/RNAemia was observed between the DENV-exposed and DENV-naïve groups [30]. This study also quantitatively measured the presence of ZIKV in urine, cerebrospinal fluid, vaginal swabs and saliva and did not find any association between flavivirus exposure history and RT-qPCR confirmed presence of ZIKV in these other compartments. Finally, the investigators noted no significant difference in clinical endpoints, histopathology, biochemistry or hematological indices measured in the DENV naïve and DENV exposed groups, although they acknowledged that a NHP model is a limited proxy for human ZIKV disease [30].

Pantoja and colleagues compared the ZIKV viral kinetics of n = 4 experimentally ZIKV H/PF/2013 inoculated rhesus macaques with a history of experimental DENV-1 or DENV-2 exposure 2.8 years prior. This was compared to a group of n = 4 ZIKV rhesus infected macaques with no prior DENV or ZIKV exposure [43]. Like McCracken et al, they too confirmed that DENV immune sera elicited ADE of a ZIKV strain *in-vitro*. Sera specimens were collected on days 1–10, 15, and 30 after ZIKV inoculation and ZIKV viremia was measured by qRT-PCR. While they observed a shorter duration of ZIKV viremia in DENV-exposed NHP (25 vs 31 viremia days), there was no statistically significant difference in peak magnitude of viremia between the DENV-experienced and DENV-naive groups, nor was there correlation between peak ZIKV titer and day minus-30 (pre-challenge) DENV sero-titers [43]. No difference in urine and saliva ZIKV PCR positivity was noted between the DENV exposed and DENV naïve groups. While a detailed comparison of cytokine levels between the two groups did suggest that prior DENV exposure modulated ZIKV cytokine profiles, this conclusion was subject to both small sample sizes and multiple statistical comparisons. This was also a consideration in comparing biochemistry, hematological and clinical markers of ZIKV infection between the DENV exposed and DENV naïve cohorts. There were only minor differences in these measurements between the two groups, and in sum were: (i) a statistically significant drop in neutrophil count at day 7 post ZIKV inoculation in the DENV exposed group, (ii) a statistically significant increase in the percent monocyte distribution at day 7 post ZIKV inoculation in the DENV exposed group, and (iii) a non-statistically significant trend toward higher ALT values one week after ZIKV inoculation in the DENV naïve group.

### Non-human primate study pooled-analysis

The NHP study by Pantoja et al and McCracken et al, in contrast to all eligible human studies, measured the same outcome (viremia/RNAemia) in the same way in ZIKV-inoculated rhesus macaques with and without known DENV exposure. As either study may have been underpowered to detect small increases in ZIKV viremia due to prior DENV exposure, we undertook a pooled-analysis to increase the effective sample size. This leveraged the 8 published data points from Pantoja et al (n = 4 DENV naïve NHP, n = 2 DENV-1 exposed NHP and n = 2 DENV-2 exposed NHP), and the 17 data points from McCracken et al (n = 12 flavivirus naïve NHP, n = 4 DENV-2 exposed NHP, n = 1 DENV-4 exposed NHP). These individual 17 data points from the McCracken et al study were not previously published as individual non-aggregated enumerated data (that is, they only presented in graphical figures) but were made available by the investigators for this pooled analysis (S2 Table). As the study protocol by McCracken et al included an *a priori* schedule of animal sacrifice before day 10 in a number of these NHPs, we only included peak viremia data in those NHP in which a peak viremia was observed (that is, declining viremia data was measured after a maximum value was observed). For this reason, we excluded n = 3 NHPs from the pooled analysis 09U038, 11U018 and M232 (see S2 Table). For the Pantoja et al study, a peak viremia was demonstrated in all n = 8 NHP. The data points from these two studies were abstracted and merged in Excel before importing into Stata version 12.0. The data entry was then double checked for data entry errors. All RNAemia data underwent log transformation to normalize the data, before examining the transformed data for residual skew. Comparison of mean and median RNAemia between the groups was performed using the two sample t-test and the Mann-Whitney U test in the Stata package. A statistical significance threshold of p = 0.05 was selected.

The 25 NHPs in this pooled dataset had a mean and median ZIKV peak RNAemia of 6.61 and 6.80 log10 genome equivalents/mL respectively, indicating mild skewness of the data after log transformation. The mean peak ZIKV RNAemia was higher in the DENV-exposed group (6.86 log_10_ genome equivalents/mL) compared to the DENV-naïve group (6.46 log_10_ genome equivalents/mL), but this of a small magnitude and was not statistically significant (p = 0.11 by one sided t-test, p = 0.22 two sided t-test). The median peak ZIKV RNAemia was also higher in the DENV-exposed group (6.88 log10 genome equivalents/mL) compared to the DENV-naïve group (6.40 log_10_ genome equivalents/mL), but this was also of a small magnitude and not statistically significant (p = 0.25 by Mann-Whitney U test).

The caveats to interpreting this pooled result include differences in ZIKV strains used, reproducibility of RNAemia/viremia measurements between the two studies and differences in time intervals between DENV and ZIKV exposure. Nevertheless, these results would be consistent with the findings seen in the eligible human studies that an increase in ZIKV viremia in those with prior DENV exposure is possible, but of a magnitude unlikely to be of major clinical relevance.

## Discussion

This systematic review found only five studies meeting pre-specified eligibility criteria, and while no meta-analysis was possible with study data published “as-is”, we were able to undertake a unique pooled analysis of the two NHP studies by publishing here extended data from McCracken et al [30]. Between this NHP pooled analysis and qualitative review of two of the three eligible human studies we have identified data suggesting that prior DENV infection may lead to greater ZIKV viremia compared to those without prior DENV infection in both humans and NHPs [41, 42]. However, this difference in magnitude is neither statistically significant nor of a magnitude which is likely to be clinically significant, so caution is required in interpreting these findings. Indeed, three eligible human studies did not indicate an increased risk of either adverse fetal outcomes, GBS or a composite “clinical severity score” in those with ZIKV infections who had previously been infected with DENV, when compared to those acute ZIKV infections with no prior DENV infection [5, 41, 42]. In addition, Terzian et al did not show a statistically significant increase in inflammatory cytokines associated with the trend toward higher median ZIKV viral loads in DENV IgG+ individuals [41].

Taken together, the current evidence from human and NHP studies suggests that prior DENV infection may cause a small rise in subsequent ZIKV viremia but that this is likely not clinically relevant. This highlights that caution is required when interpreting early in vitro and small animal model data which characterize emerging epidemic viruses, particularly during international public health emergencies, and especially because the clinical significance of in vitro and small animal model data is often unclear. This systematic review provides a cautionary tale of this common laboratory-clinical disconnect.

One major caveat in interpreting these summary findings is that that the DENV-ZIKV ADE phenomenon may be time-dependent in the same way that DENV-DENV ADE is also dependent on the interval between primary and secondary infections [28]. It may be that enhancement of ZIKV replication would be more likely to occur several years post-DENV exposure as cross-neutralizing titers of DENV wane over time [28]. The human studies identified in this systematic review did not ascribe a time dimension to prior DENV exposure and therefore may have missed an effect that is time-dependent. This could be explicitly examined should further outbreaks of ZIKV occur in the Americas or in other regions with syndemic DENV circulation that may be at risk of major ZIKV epidemics [14].

The choice of serological assay used in most of the human studies examining the role of prior DENV exposure on ZIKV outcomes was the major limitation in interpreting these data. In two of the three studies, ELISA IgG assays were used, and seroneutralization was performed in only one study. For this reason, it is difficult to infer that DENV and not some other related flavivirus infection and/or flavivirus vaccine product was the cause of the positive DENV IgG results. This could cause misclassification bias of the exposure of interest, a point which should be attended to in future studies, particularly with the availability of increasingly specific NS1 based ELISA assays which may be useful in some locations where access to the technically demanding reference plaque reduction neutralization or micro-neutralization assays may be limited [44]. Improved standardization of flavivirus serological assays, along the lines of what is being proposed for influenza, may also improve the generalization of serological results across future studies [45].

The other primary limitation in this systematic review is the low number of studies meeting eligibility criteria for inclusion, therefore limiting a quantitative meta-analysis. The paucity of eligible studies is remarkable given that the ZIKV pandemic caused over 583,000 suspected and 223,000 confirmed ZIKV autochthonous cases across 49 countries and territories in the Americas by the end of 2017 [13]. This prompts consideration of more expeditious data-sharing and collaborative research efforts to rapidly answer pertinent biological questions during emerging viral epidemics [46]. While we were unable to formally assess publication bias in a meta-analysis framework, it too could perhaps explain the lack of published studies.

Our systematic review also prompts further study into whether prior ZIKV exposure may worsen DENV clinical outcomes. The global ZIKV outbreak has resulted in a large population primed with ZIKV within DENV endemic areas and it is unclear how ZIKV exposure on a population level will shape future DENV epidemics and clinical DENV outcomes in this region, a reverse of the study question examined in this work. While existing data so far suggests that ZIKV circulation in the Americas was associated with a contemporaneous reduction in DENV cases [47], it remains to be seen whether waning ZIKV antibodies over time may subsequently cause DENV infections with higher viral loads and therefore trigger particularly large DENV epidemics, a phenomenon seen when DENV serotypes sequentially circulate through a geographic region [48]. Indeed, data exists suggesting that ZIKV itself may promote ADE during a subsequent DENV infection, with a study of NHP observing higher peak DENV-2 viremia in ZIKV exposed rhesus macaques [9]. Further studies, ideally leveraging prospective flavivirus surveillance cohorts already established in Latin America, as well as rapidly shared public health sentinel surveillance data will be critical in addressing this question which has major implications for flavivirus vaccine development programs and epidemic preparedness in the tropics [48].

### Disclaimer

The views expressed in this article are those of the authors and do not necessarily reflect the official policy or position of the Department of the Army, Department of Defense nor the U.S. Government. Several of the authors are US Government Employees. This work was prepared as part of their official duties. Title 17 U.S.C. § 105 provides that ‘Copyright protection under this title is not available for any work of the United States Government.’ Title 17 U.S.C. §101 defines a U.S. Government work as a work prepared by a military service member or employee of the U.S. Government as part of that person’s official duties.

## Supporting information

S1 Box PubMed search ontology. (DOCX)

S1 Table Quality grading tool. (DOCX)

S2 Table Extended data by McCracken et al (previously unpublished in this format). (DOCX)

S3 Table Required PRISMA checklist. (DOCX)
