# Supplementary material for: Does prior dengue virus exposure worsen clinical outcomes of Zika virus infection? A systematic review, pooled analysis and lessons learned
Source: PLoS Negl Trop Dis. 2019 Jan 25;13(1):e0007060. doi: 10.1371/journal.pntd.0007060 (PMC6370234; doi:10.1371/journal.pntd.0007060)
Supplement: S1 Box — (DOCX) [file pntd.0007060.s001.docx]

**Box S1. PubMed (MEDLINE) Search Ontology:**

((ZIKV OR zika) AND (DENV or DENV1 OR DENV2 OR DENV3 OR DENV4 OR dengue) AND ("Antibodies, Neutralizing"[Mesh] OR "Antibody-Dependent Enhancement"[Mesh] OR “antibody dependent enhancement” OR non neutralizing antibod* OR viremia OR rnaemia OR cytokine OR enhance OR enhancement OR biomarker* OR “Biomarkers”[mh] OR clinical outcome* OR microcephaly OR “fetal loss” OR malformation* OR congenital abnormalit* OR “Congenital Abnormalities”[mh] OR guillain-barre syndrome OR meningitis OR encephalitis OR myelitis OR morbidit*[tiab] OR “Morbidity”[mh] OR hospitalization OR death)) NOT (case report* [ti] OR “Case Reports”[pt] OR “Editorial”[pt])
