# Supplementary material for: Does prior dengue virus exposure worsen clinical outcomes of Zika virus infection? A systematic review, pooled analysis and lessons learned
Source: PLoS Negl Trop Dis. 2019 Jan 25;13(1):e0007060. doi: 10.1371/journal.pntd.0007060 (PMC6370234; doi:10.1371/journal.pntd.0007060)
Supplement: S1 Table — (DOCX) [file pntd.0007060.s002.docx]

| **Table S1. Quality grading tool** |  |  |  |  |
| --- | --- | --- | --- | --- |
| Quality Item | Grade and grade criteria | | | |
|  | High-Very High | Low-Moderate | Moderate-High | Unable to judge |
| Was the population, intervention, comparator group and outcome described clearly? | Described in full detail | Not described at all | Neither described in full detail nor not described at all | Unable to judge based on the information presented |
| Were the study methods presented in a reproducible way? | Described in full detail | Not described at all | Neither described in full detail nor not described at all | Unable to judge based on the information presented |
| Was the determination of prior DENV exposure valid? | Documented molecularly or dengue NS1 confirmed prior infection, history of experimental inoculation (NHP) | Patient history/clinical impression or low confidence serology^a^ | High confidence serology^b^ | Unable to judge based on the information presented |
| Was acute ZIKV exposure measured in a valid way? | Molecular confirmation (acute), known experimental inoculation (NHP) | Patient history/clinical impression or low-confidence serology^c^ | High confidence serology^d^ | Unable to judge based on the information presented |
| Were clinical outcomes and/or their laboratory proxies measured in a valid way? | Measured with high validity | Invalid method of measurement | Neither measured with high validity nor measured with an invalid method | Unable to judge based on the information presented |
| ^a^Example = DENV ELISA IgG | | | | |
| ^b^Example = DENV plaque reduction neutralization assay or microneutralization assay | | | | |
| ^c^Example = ZIKV ELISA IgM pos (acute specimen) + DENV IgM neg (acute specimen) | | | | |
