# Supplementary material for: Does prior dengue virus exposure worsen clinical outcomes of Zika virus infection? A systematic review, pooled analysis and lessons learned
Source: PLoS Negl Trop Dis. 2019 Jan 25;13(1):e0007060. doi: 10.1371/journal.pntd.0007060 (PMC6370234; doi:10.1371/journal.pntd.0007060)
Supplement: S2 Table — (DOCX) [file pntd.0007060.s003.docx]

| \| **Table S2. Extended data by McCracken et al (previously unpublished in this format)** \| \| \| \| \| \| \| \| \| \| \| \| \| \| --- \| --- \| --- \| --- \| --- \| --- \| --- \| --- \| --- \| --- \| --- \| --- \| --- \| \| Monkey ID \| Sera ZIKV titer by day post inoculation (GE/mL) \| \| \| \| \| \| \| \| \| \| DENV exposure \| Peak titer^a^ (GE/mL) \| \| 1 \| 2 \| 3 \| 4 \| 5 \| 6 \| 7 \| 8 \| 9 \| 10 \| \| 09U029 \| 0.00E+00 \| 1.80E+02 \| 2.66E+04 \| 1.06E+04 \| 9.33E+05 \| 4.41E+05 \| 4.69E+04 \| 3.25E+02 \| 0.00E+00 \| 5.77E+03 \| Naïve \| 9.33E+05 \| \| 09U038^b^ \| 1.49E+02 \| 2.22E+03 \| N/A \| N/A \| N/A \| N/A \| N/A \| N/A \| N/A \| N/A \| Naïve \| N/A \| \| 10U001 \| 0.00E+00 \| 4.12E+03 \| 1.02E+06 \| 7.90E+04 \| 4.06E+05 \| 6.01E+02 \| 0.00E+00 \| 0.00E+00 \| 0.00E+00 \| 0.00E+00 \| Naïve \| 1.02E+06 \| \| 10U003 \| 0.00E+00 \| 4.51E+03 \| 1.60E+05 \| 5.86E+05 \| 1.14E+05 \| 1.85E+03 \| 0.00E+00 \| 6.76E+02 \| 0.00E+00 \| 5.52E+01 \| Naïve \| 5.86E+05 \| \| 10U021 \| 1.21E+02 \| 1.42E+04 \| 2.81E+05 \| 7.56E+05 \| 2.63E+06 \| 6.60E+04 \| 6.54E+02 \| 0.00E+00 \| 0.00E+00 \| 0.00E+00 \| Naïve \| 2.63E+06 \| \| 10U030 \| 0.00E+00 \| 4.17E+02 \| 2.41E+03 \| 1.07E+04 \| 1.57E+04 \| 1.53E+03 \| 5.21E+02 \| 0.00E+00 \| 0.00E+00 \| 0.00E+00 \| Naïve \| 1.57E+04 \| \| 10U032 \| 0.00E+00 \| 1.61E+04 \| 1.01E+06 \| 6.86E+05 \| 2.66E+05 \| 1.08E+03 \| 1.78E+03 \| 9.21E+02 \| 3.34E+02 \| 0.00E+00 \| Naïve \| 1.01E+06 \| \| 10U036 \| 2.03E+02 \| 1.62E+03 \| 1.75E+05 \| 5.15E+05 \| 1.91E+06 \| 7.58E+03 \| 5.01E+02 \| 0.00E+00 \| 0.00E+00 \| 0.00E+00 \| Naïve \| 1.91E+06 \| \| 10U039 \| 0.00E+00 \| 8.33E+03 \| 2.91E+05 \| 1.37E+06 \| 4.93E+06 \| 3.78E+04 \| 7.78E+02 \| 3.13E+02 \| 0.00E+00 \| N/A \| Naïve \| 4.93E+06 \| \| 10U043 \| 5.65E+02 \| 1.12E+03 \| 8.03E+04 \| 1.21E+06 \| 1.17E+07 \| 1.42E+05 \| 1.97E+03 \| 3.14E+02 \| 0.00E+00 \| 0.00E+00 \| Naïve \| 1.17E+07 \| \| 10U047 \| 0.00E+00 \| 1.71E+03 \| 1.03E+05 \| 1.06E+06 \| 8.30E+06 \| 2.38E+04 \| 2.39E+03 \| 5.26E+02 \| 0.00E+00 \| 0.00E+00 \| Naïve \| 8.30E+06 \| \| 11U018^b^ \| 2.43E+02 \| 8.42E+03 \| 4.43E+05 \| 8.83E+05 \| N/A \| N/A \| N/A \| N/A \| N/A \| N/A \| Naïve \| N/A \| \| M230 \| 0.00E+00 \| 2.67E+03 \| 5.41E+04 \| 2.59E+05 \| 2.07E+06 \| 3.55E+05 \| 5.12E+03 \| 0.00E+00 \| 0.00E+00 \| 0.00E+00 \| Naïve \| 2.07E+06 \| \| M236 \| 0.00E+00 \| 3.40E+03 \| 1.46E+05 \| 5.57E+05 \| 2.45E+06 \| 2.83E+04 \| 3.64E+03 \| N/A \| N/A \| N/A \| Naïve \| 2.45E+06 \| \| 07U025 \| 3.64E+02 \| 1.18E+03 \| 6.74E+04 \| 6.01E+05 \| 7.53E+06 \| 2.23E+04 \| 7.11E+03 \| 0.00E+00 \| 0.00E+00 \| 0.00E+00 \| DENV \| 7.53E+06 \| \| M232^b^ \| 0.00E+00 \| 8.64E+03 \| N/A \| N/A \| N/A \| N/A \| N/A \| N/A \| N/A \| N/A \| DENV \| N/A \| \| 11U032 \| 4.33E+02 \| 1.27E+03 \| 4.78E+05 \| 1.18E+05 \| 3.67E+03 \| 3.64E+02 \| 4.05E+02 \| 0.00E+00 \| 0.00E+00 \| 0.00E+00 \| DENV \| 4.78E+05 \| \| 11U040 \| 4.14E+02 \| 4.47E+04 \| 2.76E+05 \| 1.51E+06 \| 5.19E+03 \| 2.70E+03 \| 0.00E+00 \| 0.00E+00 \| 0.00E+00 \| N/A \| DENV \| 1.51E+06 \| \| 11U046 \| 4.51E+02 \| 2.95E+04 \| 1.77E+05 \| 7.33E+06 \| 4.71E+05 \| 1.38E+03 \| 0.00E+00 \| 0.00E+00 \| 3.31E+02 \| 0.00E+00 \| DENV \| 7.33E+06 \| \| 10U040 \| 1.95E+02 \| 4.21E+03 \| 1.27E+06 \| 4.37E+06 \| 6.35E+06 \| 1.64E+04 \| 1.89E+03 \| 4.08E+01 \| 0.00E+00 \| 0.00E+00 \| DENV \| 6.35E+06 \|   GE/mL = genome equivalents/mL as determined by RT-PCR  ^a^Peak titer identified by maximum titer when followed by a measurement of a lower titer. In those animals which underwent early sacrifice, the peak titer was unable to be identified  ^b^Animal sacrificed early following *a priori* protocol |
| --- | --- | --- | --- | --- | --- | --- | --- | --- | --- | --- | --- | --- | --- | --- | --- | --- | --- | --- | --- | --- | --- | --- | --- | --- | --- | --- | --- | --- | --- | --- | --- | --- | --- | --- | --- | --- | --- | --- | --- | --- | --- | --- | --- | --- | --- | --- | --- | --- | --- | --- | --- | --- | --- | --- | --- | --- | --- | --- | --- | --- | --- | --- | --- | --- | --- | --- | --- | --- | --- | --- | --- | --- | --- | --- | --- | --- | --- | --- | --- | --- | --- | --- | --- | --- | --- | --- | --- | --- | --- | --- | --- | --- | --- | --- | --- | --- | --- | --- | --- | --- | --- | --- | --- | --- | --- | --- | --- | --- | --- | --- | --- | --- | --- | --- | --- | --- | --- | --- | --- | --- | --- | --- | --- | --- | --- | --- | --- | --- | --- | --- | --- | --- | --- | --- | --- | --- | --- | --- | --- | --- | --- | --- | --- | --- | --- | --- | --- | --- | --- | --- | --- | --- | --- | --- | --- | --- | --- | --- | --- | --- | --- | --- | --- | --- | --- | --- | --- | --- | --- | --- | --- | --- | --- | --- | --- | --- | --- | --- | --- | --- | --- | --- | --- | --- | --- | --- | --- | --- | --- | --- | --- | --- | --- | --- | --- | --- | --- | --- | --- | --- | --- | --- | --- | --- | --- | --- | --- | --- | --- | --- | --- | --- | --- | --- | --- | --- | --- | --- | --- | --- | --- | --- | --- | --- | --- | --- | --- | --- | --- | --- | --- | --- | --- | --- | --- | --- | --- | --- | --- | --- | --- | --- | --- | --- | --- | --- | --- | --- | --- | --- | --- | --- | --- | --- | --- | --- | --- | --- | --- | --- | --- | --- | --- | --- | --- | --- | --- | --- | --- | --- | --- | --- | --- | --- | --- | --- | --- | --- | --- | --- | --- | --- | --- | --- | --- | --- | --- | --- | --- | --- | --- | --- | --- | --- | --- | --- |
